# Ascending Aortic Strain Analysis Using 2‐Dimensional Speckle Tracking Echocardiography Improves the Diagnostics for Coronary Artery Stenosis in Patients With Suspected Stable Angina Pectoris

**DOI:** 10.1161/JAHA.118.008802

**Published:** 2018-07-07

**Authors:** Zhaohui Bu, Jun Ma, Yibo Fan, Zhiqing Qiao, Yu Kang, Ying Zheng, Wei Wang, Yongping Du, Zheng Zheng, Xuedong Shen, Ben He, Jun Pu

**Affiliations:** ^1^ Institute of Biomedical Engineering University of Shanghai for Science and Technology Shanghai China; ^2^ Department of Cardiology Ren Ji Hospital School of Medicine Shanghai Jiao Tong University Shanghai China; ^3^ Department of Cardiology Shanghai Chest Hospital Shanghai Jiao Tong University Shanghai China

**Keywords:** aortic stiffness, circumferential ascending aortic strain, coronary artery disease, 2‐dimensional speckle tracking echocardiography, Coronary Artery Disease

## Abstract

**Background:**

Arterial stiffening and atherosclerosis tend to coexist. Strain imaging, using a 2‐dimensional speckle tracking (2D‐ST) method, has been used for arterial stiffness assessment and early identification of atherosclerosis. We investigated whether the ascending aortic strain assessed by 2D‐ST echocardiography at rest can predict the presence of coronary artery disease (CAD).

**Methods and Results:**

Two hundred seventy‐one consecutive patients with suspected stable angina pectoris sequentially underwent exercise treadmill testing, 2‐dimensional echocardiography, M‐mode echocardiography, 2D‐ST echocardiography, and coronary angiography. Circumferential ascending aortic strain (CAAS) and radial ascending aortic strain were assessed by 2D‐ST echocardiography. Ninety‐two patients with coronary lumen area stenosis ≥70% were categorized as having significant CAD. Global CAAS was significantly lower in patients with significant CAD (7.41±2.30% versus 11.54±4.03%; *P*<0.001) and remained an independent predictor of significant CAD (odds ratio, 0.64 [0.54–0.75]; *P*<0.001) after multivariate regression. Based on the receiver operating characteristic curve for diagnosing significant CAD, the optimal cut‐off value of global CAAS was ≤9.22% (sensitivity, 86%; specificity, 70%; area under curve=0.82; *P*<0.001). Global CAAS decreased with increasing severity of CAD and was significantly associated with 3‐vessel disease (odds ratio, 0.58 [0.42–0.79]; *P*<0.001). Diagnostics for significant CAD were remarkably better for global CAAS combined with exercise treadmill testing than for exercise treadmill testing alone (area under curve=0.88 versus 0.78; *P*<0.001).

**Conclusions:**

Global CAAS assessed by 2D‐ST echocardiography at rest was able to predict the presence of significant CAD and identify multivessel disease. In addition, global CAAS combined with exercise treadmill testing remarkably improved the diagnostics for significant CAD.

**Clinical Perspective**

**What Is New?**

- This study demonstrated, for the first time, that global circumferential ascending aortic strain (CAAS) obtained by 2‐dimensional speckle tracking echocardiography at rest predicted significant coronary artery disease (CAD) in consecutively enrolled patients with suspected stable angina pectoris.
- Global CAAS decreased gradually with increasing severity of CAD, as determined by the increasing number of coronary vessels with lumen area stenosis ≥70%, and identified multivessel disease
- Global CAAS combined with the exercise treadmill test remarkably improved the diagnostics for significant CAD.

**What Are the Clinical Implications?**

- Global CAAS analysis based on 2‐dimensional speckle tracking echocardiography is a rapid, robust, and reproducible noninvasive method without contraindications and radiation exposure.
- Based on the present study, 2‐dimensional speckle tracking global CAAS may be a feasible and accurate tool for clinicians in the screening of patients with significant CAD.
- Furthermore, global CAAS may become a supplementary index for risk stratification of CAD, given that it is capable of identifying multivessel CAD.

## Introduction

Arterial stiffening is one of the earliest detectable manifestations of adverse structural and functional changes within the vessel wall.^1^ Degenerative stiffening of the arterial beds (ie, arteriosclerosis) and atherosclerosis tend to coexist, causing progressive, diffuse, and age‐related deterioration in all vascular beds.^1^, ^2^ Increased aortic stiffness is a risk factor for cardiovascular diseases and a predictor of cardiovascular morbidity and mortality.^3^, ^4^, ^5^, ^6^ Consequently, assessment of arterial stiffness is increasingly used in clinical practice. However, validity and reproducibility of the conventional methods used for local assessment of arterial stiffness, such as elastic modulus, distensibility, and stiffness index, are limited by their dependence on the patient's blood pressure.^7^, ^8^, ^9^

Two‐dimensional (2D) strain echocardiography was developed to allow a rapid, accurate, angle‐independent determination of regional myocardial deformation.^10^ Circumferential deformation of the descending thoracic aorta,^11^ abdominal aorta,^12^, ^13^ or carotid arteries^14^ can be measured using 2D speckle tracking (2D‐ST), allowing a simple and accurate determination of aortic stiffness.^15^ An association between circumferential strain and the progression of vascular stiffness with age has been reported by Bjällmark et al.^16^ The investigators demonstrated that strain imaging, using the 2D‐ST technique, is a sensitive and reliable method for the assessment of elastic properties in the common carotid artery and offers a valuable tool for early identification of patients with atherosclerotic disease. Kim et al reported that a reduced carotid strain value measured using the 2D‐ST technique was associated with the presence and severity of coronary artery disease (CAD).^9^

Aorta influences the circulation in a global fashion by serving as a conduit and playing important roles in modulating left ventricular (LV) performance, myocardial perfusion, central hemodynamics, and arterial function throughout the entire cardiovascular system.^17^ The elastic properties of the aorta can be related to the degree of CAD.^18^ Hence, it would be appealing if the ascending aortic strain assessed by 2D‐ST echocardiography could improve the diagnostics for coronary artery stenosis.

Chest pain, a potential manifestation of CAD, is a common complaint of patients presenting to cardiology departments.^19^ Exercise treadmill testing (ETT) is a recommended noninvasive diagnostic test for detection of CAD in patients with chest pain (chest discomfort) syndromes or potential symptom equivalents^20^; however, its limited sensitivity and specificity have been highlighted.^21^ The purpose of the present study in consecutively enrolled patients with suspected stable angina pectoris (SAP) was to investigate whether ascending aortic strain assessed by 2D‐ST echocardiography at rest predicts the presence of significant CAD and is related to the severity of CAD. Additionally, we also investigated whether ascending aortic strain combined with ETT can significantly improve the diagnostics for CAD.

## Methods

Data, analytical methods, and study materials will be made available to other researchers for purposes of reproducing the results or replicating the procedure. This material can be made available by the first author and the corresponding author on reasonable request.

### Study Population

A total of 308 consecutive patients with suspected SAP were enrolled from January 2015 to May 2017. SAP was defined as chest pain or discomfort (angina) suspected to be caused by myocardial ischemia.^21^, ^22^ Anginal symptoms were considered stable if they had been occurring for several weeks without deterioration and were typically induced by activity or stress.^22^ Personal information and clinical data, including age, sex, weight, height, blood pressure, past medical history, smoking status, family history, and medication, were obtained from patient interviews. Fasting blood samples for glucose and cholesterol were collected at the first interview. Exclusion criteria included known CAD, valvular heart disease, heart dysfunction determined by LV ejection fraction <50%, arrhythmias, intraventricular conduction disturbances, pathological Q waves, chronic kidney disease, aortic aneurysms, and systematic diseases affecting the aorta such as Marfan syndrome and Takayasu's arteritis.^23^

All patients underwent ETT, 2D echocardiography, M‐mode echocardiography, and 2D‐ST echocardiography before coronary angiography. Coronary angiography was performed regardless of the outcome of echocardiography and ETT. The study was approved by the institutional review board, and all patients provided written informed consent.

### Exercise Treadmill Test

Symptom‐limited ETT was performed according to the recommended Bruce treadmill protocol.^20^ Heart rate, blood pressure, and 12‐lead ECGs were recorded at rest and during each minute of exercise. ECGs were analyzed by an investigator using a Mortara X‐Scribe II system (Mortara Instruments, Milwaukee, WI). Criteria for test positivity included the following: (1) horizontal or downsloping ST‐segment depression ≥1 mm (0.1 mv) at 60 ms after the J point and (2) ST‐segment elevation ≥1 mm at 60 ms after the J point.^20^ Duke Treadmill Score (DTS) is a weighted combination of exercise duration, ST‐segment deviation, and the presence and nature of angina during testing. In the present study, DTS was used as the outcome of ETT and calculated as follows: DTS=exercise time (minutes)−5×maximal ST‐segment deviation during or after exercise (millimeters)−4×angina score (no angina=0, nonlimiting angina=1, and test‐limiting angina=2).^24^

### Echocardiographic Imaging

Transthoracic echocardiography was performed using a Vivid E9 ultrasound system with a 3.5‐MHz transducer (GE, Milwaukee, WI) after ETT and before coronary angiography. The operator was blinded to the patient history and results of ETT. Before echocardiography, blood pressure was measured at the right brachial artery using an electronic sphygmomanometer (Omron HBP‐1100U; Omron Instruments, Kyoto, Japan) with the patient supine. Standard 2D echocardiographic images were obtained during breathhold when patients were in the left lateral decubitus position. A frame rate of 55 to 75 frames per second was used for 2D speckle tracing analysis.

### Conventional Echocardiography

Each echocardiographic measurement was taken from the average of 3 continuous cardiac cycles. Conventional echocardiographic values, including LV dimensions, LV ejection fraction, LV fractional shortening, LV mass index, peak transmitral early velocity, peak atrial diastolic filling, deceleration time, peak longitudinal early diastolic velocity, and peak transmitral early diastolic inflow velocity/peak longitudinal early diastolic velocity ratio, were measured and calculated according to the recommendations of the American Society of Echocardiography.^25^

### M‐Mode Echocardiography

**Figure 1:**
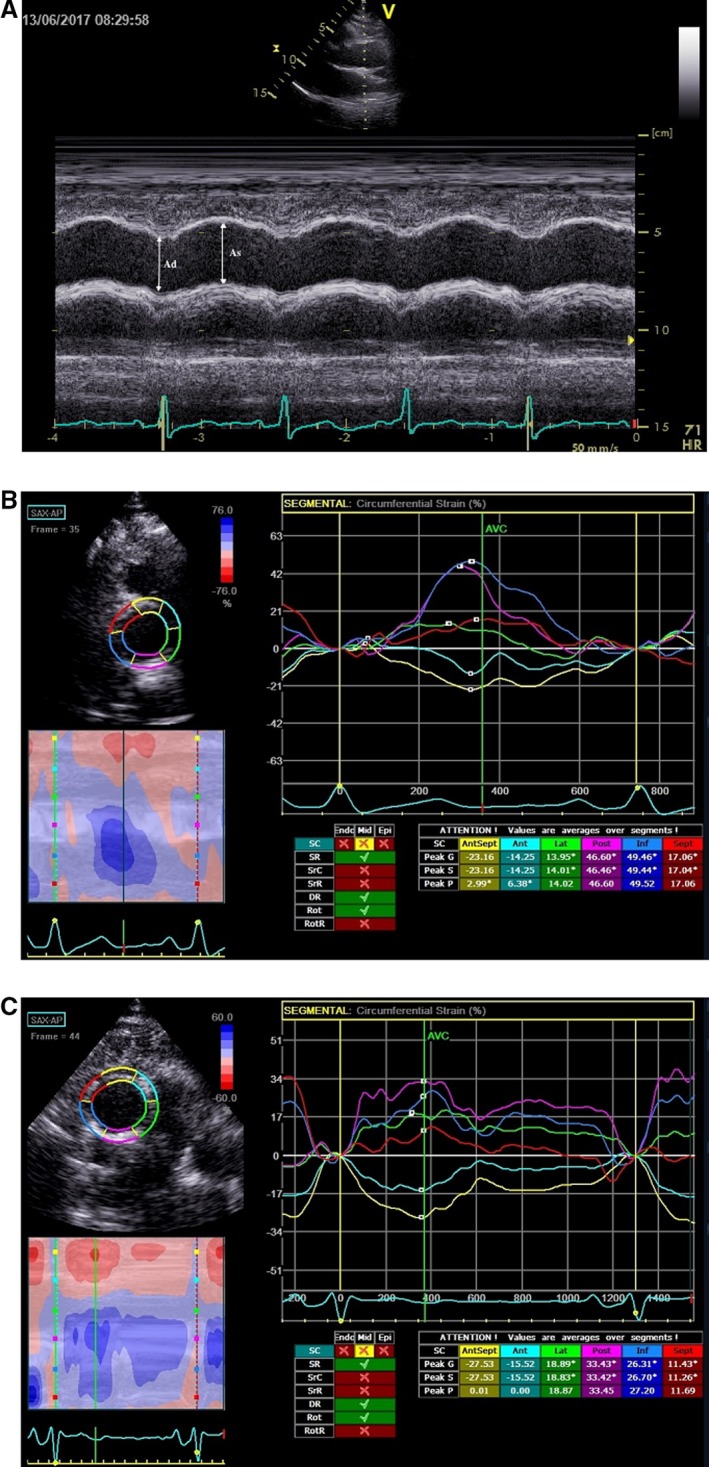
A, M‐mode 2D‐guided aortic image obtained from the aortic long‐axis view. A_s_, ascending aortic diameter in systole; A_d_, ascending aortic diameter in diastole. B, Representative CAAS curves in a patient without significant coronary stenosis. Peak values of CAAS in each color‐coded curve were measured, and then the global CAAS was calculated as the mean of 6 peak values. This patient had a global CAAS of 14.94%. C, Representative CAAS curves in a patient with significant coronary stenosis. This patient had a global CAAS of 7.8%. 2D indicates 2‐dimensional; CAAS, circumferential ascending aortic strain.

Ascending aorta was imaged at 3 cm above the aortic valve from the parasternal long‐axis view^26^ (Figure 1A). Systolic aortic diameter (A_s_) was measured at the point of maximal anterior motion of the ascending aorta (systole), and diastolic aortic diameter (A_d_) was measured at the q wave on ECG (end diastole) using M‐mode echocardiography. The mean of 5 measurements for diameter in sequential cardiac cycles was used for data analysis. Three elastic indices of aortic stiffness, namely aortic distensibility (D), stiffness index (β), and elastic modulus (E_p_), were calculated as D=2(A_s_−A_d_)/[A_d_(P_s_−P_d_)], β=ln(P_s_/P_d_)/[(A_s_−A_d_)/A_d_], and E_p_=(P_s_−P_d_)/[(A_s_−A_d_)/A_d_], respectively, where P_s_=systolic blood pressure, P_d_=diastolic blood pressure, and ln=natural logarithm.^26^

### 2D‐ST Echocardiography

At the same sampling location of M‐mode echocardiography measurements, a circular plane of the ascending aorta was obtained at 3 cm above the aortic valve from the parasternal short‐axis view. 2D image acquisition was performed at end‐expiratory apnea. Loops of 3 cardiac cycles were stored digitally and analyzed offline using commercial software (EchoPAC; GE Healthcare, Horten, Norway).

Ascending aortic strain was assessed by 2D‐ST echocardiography. In high‐frame‐rate images of ascending aorta, a loop was manually drawn along the inner edge of the aortic wall, and then an additional loop near the outer edge of the aortic wall was automatically generated by the software. Because of the small thickness of the aortic wall, the width of the region of interest was adjusted to the minimum allowed by the software.^27^ Before processing, the operator visually checked, or the software automatically determined, whether the internal loop followed the aortic inner side throughout the cardiac cycle. If the tracking was suboptimal, manual adjustments of region‐of‐interest size were performed.

The software automatically divided the optimal aortic wall image into 6 equally sized color‐coded segments (yellow, light blue, green, purple, blue, and red). Quantitative curves with different colors were used to represent the 2D‐ST variables of different segments of aortic wall. Peak values of circumferential strain and radial strain in each segment, which typically appeared near the aortic valvular closure (systole), were identified (Figure 1B and 1C). A global circumferential ascending aortic strain (CAAS) was calculated as the mean value of peak CAAS of the 6 segments. A corrected CAAS was calculated as the global CAAS/pulse pressure, according to Yuda et al.^14^ A global radial ascending aortic strain (RAAS) was calculated as the mean value of peak RAAS of the 6 segments.

### Coronary Angiography

Study participants were subjected to coronary angiography, which was performed by the percutaneous radial or femoral approach. An experienced operator blinded to the results of the preceding examinations analyzed the angiograms visually. Significant coronary stenosis was defined as ≥70% reduction in the coronary lumen area.

### Statistical Analysis

Normality of the distribution of continuous variables was confirmed by the Kolmogorov–Smirnov test. Continuous variables are presented as the mean±SD, and the Student *t* test was used to compare the differences between groups. Categorical variables are expressed as frequencies and percentages and were compared using the chi‐squared test or Fisher's exact test. Multiple step‐wise logistic regression was performed to assess the influence of baseline characteristics, including age, sex, body mass index (BMI), heart rate, hypertension, diabetes mellitus, hypercholesterolemia, smoking, pulse pressure on ascending aortic strain, and stiffness parameters. For the optimal predictors, receiver operating characteristic (ROC) curves were constructed, and the area under the curve (AUC) was calculated. The difference in AUC from ROC curves was evaluated by the Delong method. Subsequently, the optimal cut‐off value of the predictors with the highest sensitivity and specificity was selected using ROC curves. One‐way ANOVA was used to assess whether the aortic strain value changed with increasing severity of CAD, as determined by an increasing number of stenotic coronary vessels. The Student–Newman–Keuls method was used for post‐hoc tests of one‐way ANOVA. A two‐tailed *P*<0.05 was considered statistically significant. Inter‐ and intraobserver reproducibility for measurement of global CAAS and global RAAS were assessed on recorded images from 30 randomly selected patients with the Bland–Altman method by repeating the entire processes from the initial 2D image acquisition of ascending aorta. Data were analyzed using SPSS (version 23.0; SPSS, Inc, Chicago, IL) and MedCalc software (version 15.2.0; MedCalc Software bvba, Ostend, Belgium).

## Results

Among the 308 consecutively enrolled patients, 37 were excluded because of poor echocardiographic image quality. Of the remaining 271 patients, 92 were categorized as having significant CAD with coronary area stenosis ≥70%, whereas 179 had nonsignificant CAD or no CAD.

Baseline characteristics are summarized in Table 1. Compared with patients without significant CAD, patients with significant CAD had higher age, male proportion, BMI, and prevalence of smoking. Prevalence rate of hypertension was similar between patients with and without significant CAD. Hypertension was well controlled in all patients. In patients with significant CAD, 44 (47.8%), 23 (25%), and 25 (27.2%) had 1, 2, and 3 significantly stenotic coronary vessels, respectively, as confirmed by coronary angiography. There was significant left anterior descending artery, left circumflex coronary artery, right coronary artery, and left main coronary artery stenosis in 72, 48, 45, and 6 patients, respectively.

**Table 1:** Baseline Characteristics of the Study Population

	All Patients	Patients With Significant CAD	Patients Without Significant CAD	*P* Value^a^
n (%)	271	92 (34)	179 (66)	···
Age, y	57.04±9.65	58.91±10.10	56.08±9.28	0.022
Men, n (%)	158 (58)	70 (76)	84 (47)	<0.001
Heart rate (bpm)	72.96±9.35	74.40±10.87	72.22±8.40	0.093
SBP, mm Hg	136.19±13.49	136.65±14.56	135.96±12.95	0.690
DBP, mm Hg	76.64±9.14	77.65±7.35	76.13±9.92	0.156
PP, mm Hg	59.55±15.82	59.01±15.72	59.83±15.91	0.684
Hypertension, n (%)	125 (46)	43 (47)	82 (46)	0.884
ACEI or ARB, n (%)	48 (18)	18 (20)	30 (17)	0.567
CCB, n (%)	91 (34)	36 (39)	55 (31)	0.165
β‐blocker, n (%)	34 (13)	12 (13)	22 (12)	0.859
BMI (kg/m^2^)	24.69±2.43	25.13±2.26	24.46±2.49	0.032
Diabetes mellitus, n (%)	33 (12)	15 (16)	18 (10)	0.136
Hypercholesterolemia, n (%)	44 (16)	16 (17)	28 (16)	0.712
LDL‐C (mmol/L)	2.85±0.83	2.83±1.13	2.85±0.68	0.910
HDL‐C (mmol/L)	1.32±0.41	1.26±0.29	1.34±0.44	0.198
Smoker, n (%)	82 (30)	42 (46)	40 (22)	<0.001

Continuous data are presented as mean±SD and categorical data as number (percentage). ACEI indicates angiotensin‐converting enzyme inhibitor; ARB, angiotensin receptor inhibitor; BMI, body mass index; CAD, coronary artery disease; CCB, calcium‐channel blocker; DBP, diastolic blood pressure; HDL‐C, high‐density lipoprotein cholesterol; LDL‐C, low‐density lipoprotein cholesterol; PP, pulse pressure; SBP, systolic blood pressure.

a Comparison between patients with and without significant CAD.

In patients with significant CAD, exercise performance was significantly impaired in all parameters, including exercise time (5.84±1.52  versus 7.20±1.98 minutes; *P*<0.001), exercise capacity (6.76±1.42 versus 8.36±1.79 metabolic equivalents; *P*<0.001), maximum heart rate (131.94±11.24 versus 151.15±17.74 beats per minute; *P*<0.001), maximal ST‐deviation (3.18±0.88 versus 2.31±0.97 mm; *P*<0.001), and DTS (−2.83±4.61 versus 2.13±4.77; *P*<0.001).

### Echocardiographic Measurements

Echocardiographic measurements in study participants are depicted in Table 2. In patients with significant CAD, LV diastolic function was impaired and LV mass index was higher. In terms of M‐mode–derived indices of aortic stiffness, β and E_p_ were higher, and D was lower, in patients with significant CAD (Table 2).

**Table 2:** Echocardiographic Measurements

	Patients With Significant CAD	Patients Without Significant CAD	*P* Value^a^
Conventional echocardiographic parameters			
LVEF, %	64.52±4.93	66.58±5.16	0.082
FS, %	34.39±4.15	36.10±6.72	0.270
LVMI, g/m^2^	92.69±21.24	82.02±15.06	0.019
DT, ms	189.03±48.18	181.54±32.29	0.284
E, m/s	0.70±0.16	0.74±0.15	0.063
A, m/s	0.75±0.13	0.70±0.16	0.019
E/A	0.97±0.29	1.07±0.31	0.013
e′, cm/s	10.41±2.22	11.66±2.80	0.003
E/e′	6.78±1.40	6.42±1.25	0.034
Ascending aortic stiffness parameters			
A_s_, cm	3.47±0.46	3.59±0.39	0.037
A_d_, cm	3.13±0.39	3.16±0.42	0.388
D, cm^2^dyne^−1^10^−6^	3.94±1.43	4.52±2.48	0.015
β	5.17± 3.62	4.31 ±2.47	0.041
E_p_, kPa	68.82±35.72	60.93±24.47	0.046
Ascending aortic strain			
Global CAAS, %	7.41±2.30	11.54±4.03	<0.001
Corrected CAAS, %/mm Hg	0.154±0.046	0.202±0.062	<0.001
Global RAAS, %	−20.58±9.03	−24.33±13.74	0.029

A indicates peak transmitral late diastolic inflow velocity; A_d_, diastolic ascending aortic diameter; A_s_, systolic ascending aortic diameter; CAAS, circumferential ascending aortic strain; D, aortic distensibility; DT, deceleration time of early diastolic transmitral inflow; E, peak transmitral early diastolic inflow velocity; e′, peak longitudinal early diastolic velocity obtained from the septal and lateral myocardial segment; E_p_, pressure‐strain elastic modulus; FS, left ventricular fractional shortening; LVEF, left ventricular ejection fraction; LVMI, left ventricular mass index; RAAS, radial ascending aortic strain; β, stiffness index.

a Comparison between patients with and without significant CAD.

Table 2 further shows that the global CAAS assessed by 2D‐ST echocardiography at rest was significantly lower in patients with significant CAD than in patients without CAD (7.41±2.30% versus 11.54±4.03%; *P*<0.001). Corrected CAAS was significantly reduced in patients with significant CAD (0.154±0.046%/mm Hg versus 0.202±0.062%/mm Hg; *P*<0.001). Statistical difference of the global RAAS, assessed by 2D‐ST echocardiography (−20.58±9.03% versus −24.33±13.74%; *P*=0.029), was found between patients with and without significant CAD.

### Analysis of CAAS

Table 3 lists the results of uni‐ and multivariate logistic regression analyses performed to weight the value of ascending aortic stain and stiffness parameters in predicting significant CAD. After adjusting for baseline characteristics, including age, sex, BMI, heart rate, hypertension, diabetes mellitus, hypercholesterolemia, smoking, and pulse pressure, only global CAAS (odds ratio [OR], 0.64 [0.54–0.75]; *P*<0.001) and corrected CAAS (OR, 0.80 [0.73–0.87]; *P*<0.001) remained independent predictors of significant CAD.

**Table 3:** Logistic Regression Analysis of Ascending Aortic Stiffness Parameters and Strain Values in the Presence of Significant CAD

Variables	Univariate Analysis			Multivariate Analysis^a^		
	OR (95% CI)		*P* Value	OR (95% CI)		*P* Value
β	1.10	(1.01–1.20)	0.023	1.07	(0.97–1.18)	0.198
D	0.88	(0.78–0.99)	0.040	0.87	(0.75–1.01)	0.064
E_p_	1.01	(1.00–1.02)	0.036	1.01	(0.99–1.02)	0.349
Global RAAS	1.03	(1.00–1.05)	0.021	1.03	(0.99–1.07)	0.072
Global CAAS	0.62	(0.55–0.71)	<0.001	0.64	(0.54–0.75)	<0.001
Corrected CAAS	0.85	(0.80–0.90)	<0.001	0.80	(0.73–0.87)	<0.001

CAAS indicates circumferential ascending aortic strain; CAD, coronary artery disease; CI, confidence interval; OR, odds ratio; RAAS, radial ascending aortic strain.

a Multiple stepwise logistic regression was adjusted for baseline characteristics, including age, sex, body mass index, heart rate, hypertension, diabetes mellitus, hypercholesterolemia, smoking, pulse pressure on ascending aortic strain, and stiffness parameters.

**Figure 2:**
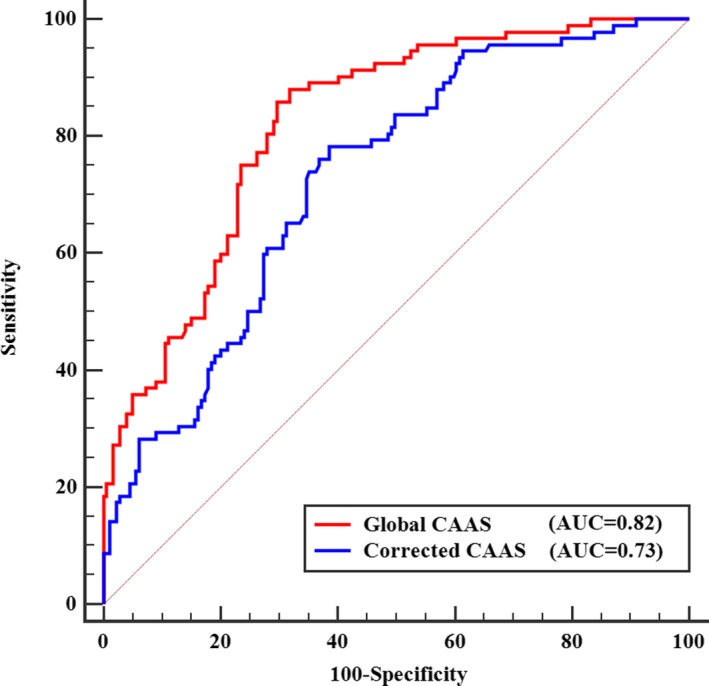
ROC curves of global CAAS and corrected CAAS for predicting significant CAD. Red line represents the ROC curve for global CAAS. Blue line represents the ROC curve for corrected CAAS. AUC indicates area under ROC curve; CAAS, circumferential ascending aortic strain; CAD, coronary artery disease; ROC, receiver operating characteristic.

Area under the ROC curve of global CAAS for diagnosing significant CAD was 0.82 (95% confidence interval [CI; 0.77–0.87]; *P*<0.001), which determined the diagnostic performance of global CAAS (Figure 2). According to the analysis of the ROC curve, the optimal cut‐off value of global CAAS was ≤9.22% with a sensitivity of 86% and a specificity of 70% (Table 4). The area under the ROC curve of the corrected CAAS for diagnosing significant CAD was 0.73 (95% CI [0.67–0.78]; *P*<0.001; Figure 2). The optimal cut‐off value for corrected CAAS (less than 0.184%/mm Hg) had a sensitivity of 78% and a specificity of 61%.

**Table 4:** Sensitivity, Specificity, Positive Predictive Value, and Negative Predictive Value of the Predictors of Significant CAD

Cut‐Off Value	Sensitivity	Specificity	PPV	NPV
Global CAAS ≤9.22%	79/92 (86%)	126/179 (70%)	79/132 (60%)	126/139 (91%)
DTS ≤−1.12	66/92 (72%)	133/179 (74%)	66/112 (59%)	133/159 (84%)
Global CAAS ≤9.22% or DTS ≤−1.12	84/92 (91%)	91/179 (51%)	84/172 (49%)	91/99 (92%)
Global CAAS ≤9.22% and DTS ≤−1.12	61/92 (66%)	168/179 (94%)	61/72 (85%)	168/199 (84%)

CAAS indicates circumferential ascending aortic strain; CAD, coronary artery disease; DTS, Duck Treadmill Score; NPV indicates negative predictive value; PPV, positive predictive value.

### Relationship Between CAAS and the Severity of CAD

**Figure 3:**
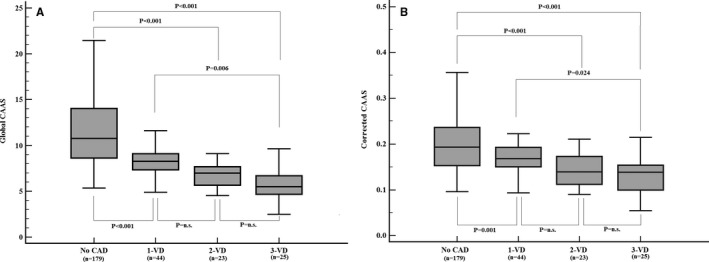
A, Global CAAS and severity of CAD. B, Corrected CAAS and severity of CAD. CAAS indicates circumferential ascending aortic strain; CAD, coronary artery disease; n.s., no significant difference; VD, vessel disease.

Global CAAS decreased incrementally with increasing severity of CAD, as determined by an increasing number of coronary vessels with lumen area stenosis ≥70%. In patients having no CAD or 1‐, 2‐, and 3‐vessel disease (VD), global CAAS was 11.54±4.03%, 8.45±2.02%, 6.91±1.54%, and 6.03±2.52% (F=31.11; *P*<0.001), respectively. In post‐hoc tests, global CAAS of patients without CAD was significantly higher than that of those with 1‐, 2‐, or 3‐VD (All *P*<0.001; Figure 3A). Global CAAS of patients with 3‐VD was significantly lower than that of those with 1‐VD (*P*=0.006; Figure 3A). Corrected CAAS also declined incrementally with increasing severity of CAD (corrected CAAS, 0.202± 0.062%/mm Hg versus 0.170±0.042%/mm Hg versus 0.141±0.037%/mm Hg versus 0.138±0.053%/mm Hg; *P*<0.001 for patients with no CAD or 1‐, 2‐, and 3‐VD, respectively; Figure 3B).

**Figure 4:**
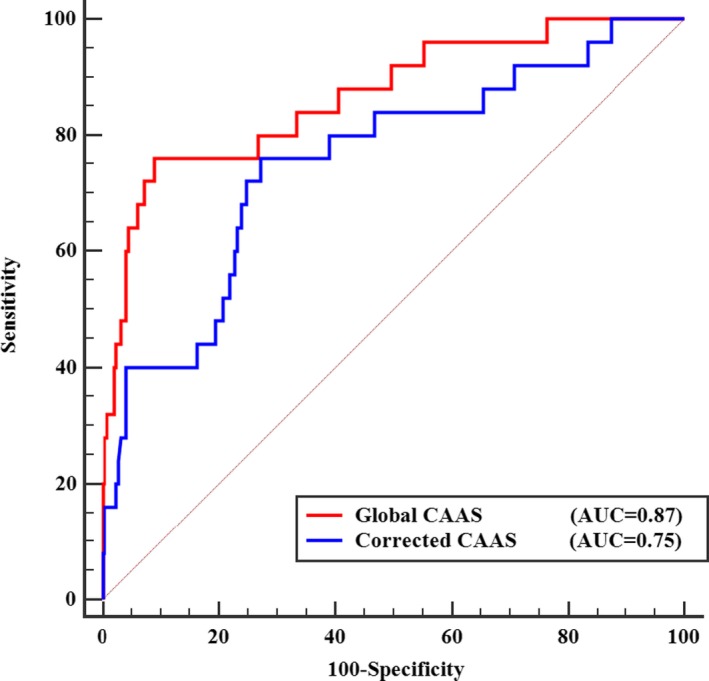
ROC curves of global CAAS and corrected CAAS for identifying 3‐vessel CAD. Red line represents the ROC curve for global CAAS. Blue line represents the ROC curve for corrected CAAS. AUC indicates area under ROC curve; CAAS, circumferential ascending aortic strain; CAD, coronary artery disease; ROC, receiver operating characteristic.

Multivariate logistic regression analysis for 3‐VD among age, sex, BMI, heart rate, hypertension, diabetes mellitus, hypercholesterolemia, smoking, pulse pressure, aortic stiffness parameters, and ascending aortic strain showed that only global CAAS (OR, 0.58 [0.42–0.79]; *P*<0.001) and corrected CAAS (OR, 0.77 [0.66–0.89]; *P*<0.001) were significantly associated with 3‐VD. The area under the ROC curve of global CAAS and corrected CAAS for detecting 3‐VD was 0.87 (95% CI [0.82–0.91]; *P*<0.001) and 0.75 (95% CI [0.70–0.80]; *P*<0.001), respectively (Figure 4). The optimal cut‐off value for global CAAS was 6.35% with a sensitivity of 76% and a specificity of 91%. The optimal cut‐off value for corrected CAAS was 0.149%/mm Hg with a sensitivity of 76% and a specificity of 73%.

### Diagnostic Performance of Global CAAS Combined With ETT

**Figure 5:**
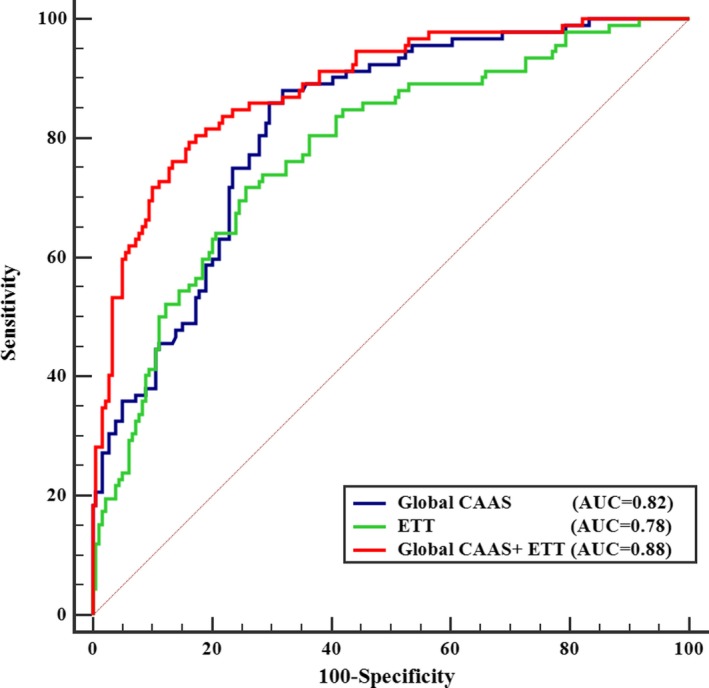
ROC curves for predicting significant CAD. Blue line represents the ROC curve for global CAAS. Green line represents the ROC curve for exercise treadmill testing (ETT), whose outcome is presented as Duck Treadmill Score (DTS). Red line represents the ROC curve for the global CAAS combined with ETT. AUC indicates area under ROC curve; CAAS, circumferential ascending aortic strain; CAD, coronary artery disease; ROC, receiver operating characteristic.

DTS was used to evaluate the ETT. After multivariate logistic regression analysis, DTS was associated with the presence of significant CAD (OR, 0.81 [0.75–0.89]; *P*<0.001). Based on the ROC curve of DTS for diagnosing significant CAD, the optimal cut‐off value of DTS was ≤−1.12 (sensitivity, 72%; specificity, 74%; Table 4). Global CAAS combined with ETT yielded a higher diagnostic power as reflected by a significantly greater AUC compared with that for ETT alone (0.88 [0.84–0.92] versus 0.78 [0.73–0.83]; *P*<0.001; Figure 5). When combining the 2 cut‐off values of global CAAS and DTS, diagnostic accuracy for significant CAD was markedly improved (Table 4).

### Intra‐ and Interobserver Variability in Strain Measurements

**Figure 6:**
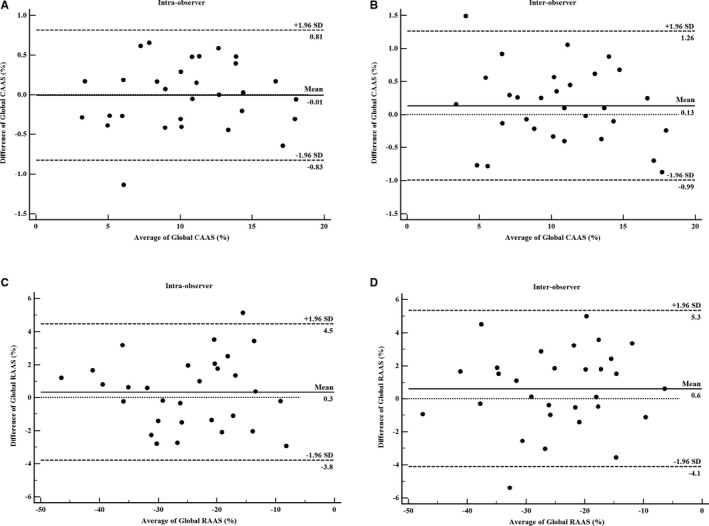
Bland–Altman analysis for: intraobserver variability of global CAAS measurement (A) and global RAAS measurement (C); and for interobserver variability of global CAAS measurement (B) and global RAAS measurement (D). Mean indicates mean difference. CAAS indicates circumferential ascending aortic strain; RAAS, radial ascending aortic strain.

A reasonable reproducibility for strain measurement using 2D‐ST echocardiography with a small bias was shown by Bland–Altman analysis (Figure 6). Intraobserver variability of global CAAS was −0.01%, and the 95% limits of agreement ranged from −0.83% to 0.81%. Interobserver variability of global CAAS was 0.13%, and the 95% limits of agreement ranged from −0.99% to 1.26%. For global RAAS, intraobserver variability was 0.3% (95% limits of agreement, −3.8–4.5%) and interobserver variability was 0.6% (95% limits of agreement, −4.1–5.3%).

## Discussion

In the present study: (1) global CAAS obtained by 2D‐ST echocardiography had a high feasibility and satisfactory reproducibility; (2) global CAAS at rest predicted significant CAD with high sensitivity (86%) in patients with suspected SAP (Tables 3 and 4); (3) global CAAS decreased gradually with increasing severity of CAD, as determined by the increasing number of coronary vessels with lumen area stenosis ≥70%, and identified multivessel disease (Figures 3 and 4); (4) corrected CAAS also predicted significant CAD and identified multivessel disease; and (5) global CAAS combined with the ETT remarkably improved diagnostics for significant CAD (Figure 5 and Table 4).

Many studies have tried to present arterial stiffness as a manifestation of atherosclerotic cardiovascular disease.^3^, ^4^, ^28^ All‐cause and cardiovascular mortality have been noted to be correlated with arterial stiffness.^3^, ^6^ Several mechanisms may explain the association between aortic stiffness and coronary heart disease. Arterial stiffening leads to an increase in central systolic blood pressure and a decrease in diastolic blood pressure.^29^ Elevation of systolic blood pressure increases myocardial oxygen demand and ventricular load, thereby inducing LV hypertrophy.^30^ Diminished diastolic blood pressure reduces coronary perfusion, resulting in subendocardial ischemia.^31^ Subsequently, a raised pulse pressure may induce arterial remodeling, increasing wall thickness, and development of plaques.^32^

Assessment of arterial stiffness is increasingly used in clinical practice. Elastic modulus, distensibility, and stiffness index β are the methods used for a local assessment of arterial stiffness.^33^, ^34^ However, validity and reproducibility of these methods are limited because of their dependence on the patient's blood pressure.^7^, ^9^ In contrast, strain imaging, using 2D ultrasonography with a speckle tracking technique, is a sensitive and reliable method for the assessment of elastic properties of arteries and offers a valuable tool for early identification of atherosclerosis.^16^ This method provides 2D data that enable researchers to assess the elastic properties of the arterial wall directly^9^, ^16^, ^27^, ^35^ and show a high sensitivity and good reproducibility.^16^, ^27^ Furthermore, the 2D‐ST method can detect heterogeneous motion pattern and local variations in compliance in different sectors of the vascular wall.^16^ In our study, global CAAS obtained by 2D‐ST echocardiography was performed over the entire circumference of the short‐axis section with high feasibility and satisfactory reproducibility. Additionally, strain is the resulting deformation (percentage change in length) of an object/material subjected to a stress force.^1^ In the case of equal arterial stiffness, a high pulse pressure should theoretically determine higher strain values.^27^ Thus, a corrected CAAS, expressed as the global CAAS/pulse pressure, was also considered in the present study.

Meanwhile, the increase in arterial stiffness was considered to be a process that can influence arterial deformation, but may not be necessarily in a homogenous way. Bjällmark et al reported that the possible heterogeneity of the deformation pattern may be further accentuated by atherosclerotic plaque and plaque calcification.^16^ In agreement with previous studies,^15^, ^16^ CAAS measurement using 2D‐ST echocardiography in the present study demonstrated the heterogeneous motion pattern and local variations in compliance of different segments of the ascending aortic wall (Figure 1B and 1C). According to previous investigators,^14^, ^15^ we divided the ascending aortic wall into 6 equally sized segments. A global CAAS was calculated as the mean value of peak CAAS of the 6 segments.

Several studies have provided evidence that elastic properties of large arteries are impaired in the presence of atherosclerotic cardiovascular disease and several risk factors.^18^ Güngör et al demonstrated that the early diastolic velocity of the anterior wall of the ascending aorta measured with pulse‐wave tissue Doppler imaging echocardiography correlates with arterial stiffening and is decreased in patients with premature CAD.^36^ However, the tissue Doppler imaging technique is angle dependent and is influenced by tethering or translational motion.^15^ The 2D‐ST technique not only avoids the limitations mentioned above, but also allows the measurement of deformation in all segments of the vessel. It has been reported, in a previous study, that a reduced carotid strain value measured using the 2D‐ST technique was associated with presence and severity of CAD.^9^ In our study, we demonstrated, for the first time, that the global CAAS assessed by 2D‐ST echocardiography can be used to predict significant CAD and identify multivessel disease in patients with suspected SAP.

Our study demonstrated that both global CAAS and corrected CAAS were significantly associated with prevalence of significant coronary stenosis. Although CAD patients were older and had a higher male proportion, BMI, and prevalence of smoking, only global CAAS and corrected CAAS remained independent predictors of significant CAD after adjustment for baseline characteristics in multivariate regression analysis (Table 3). Based on the ROC curve of the global CAAS for diagnosing significant CAD, the area under the ROC curve was 0.82 (95% CI [0.77–0.87]; *P*<0.001) and the optimal cut‐off value of global CAAS was 9.22%. The ability of global CAAS to differentiate significant CAD was remarkable, with 86% of enrolled patients with global CAAS ≤9.22% having significant coronary stenosis confirmed by coronary angiography (Table 4). According to our data, global CAAS had a high accuracy to predict significant CAD, rendering it a potential marker for CAD. Meanwhile, the area under the ROC curve of the corrected CAAS for diagnosing significant CAD was 0.73 (95% CI [0.67–0.78]; *P*<0.001; sensitivity, 78%; specificity, 61%). The fact that blood pressure and CAAS for calculation of corrected CAAS were measured at different locations imposes limitations on the diagnostic accuracy of corrected CAAS. Additionally, RAAS, based on thinning of the aortic wall, was difficult to measure accurately using 2D‐ST.^2^, ^16^ Therefore, global RAAS showed a greater variability than global CAAS and failed to become an independent predictor of significant CAD after multivariate regression in the present study.

Risk stratification is essential for patients with established coronary atherosclerosis. Our study population consisted of patients with no previous history of heart disease, a relatively low prevalence of cardiovascular risk factors, and normal LVEF determined by echocardiography. Accordingly, overall prevalence of significant CAD was low (34%). In such a low‐risk study population, the fact that global CAAS and corrected CAAS were independent predictors of significant CAD implies that global CAAS and corrected CAAS might be useful for risk stratification of patients with suspected SAP. Our study demonstrated that both global CAAS and corrected CAAS decreased incrementally with increasing severity of CAD, as determined by an increasing number of diseased vessels (Figure 3). Further analysis showed that global CAAS had a significant association with 3‐VD and was able to detect or exclude multivessel CAD with a satisfactory diagnostic performance (AUC=0.87; *P*<0.001; sensitivity, 76%; specificity, 91%; Figure 4). Corrected CAAS was also capable of detecting multivessel CAD (AUC=0.75; *P*<0.001; Figure 4). Hence, global CAAS and corrected CAAS may become supplementary indices for risk stratification of CAD.

In the conventional diagnostic procedure for CAD, ETT is a recommended diagnostic examination for patients with suspected SAP induced by coronary artery stenosis, despite its flawed specificity and sensitivity.^21^ In the present study, the sensitivity and specificity of ETT for detecting significant CAD were 72% and 74%, respectively. These values are unsatisfactory in clinical practice, where 26% of patients with normal coronary artery or nonsignificant coronary stenosis might undergo an unnecessary invasive coronary angiography. Thus, there is a clinical demand for combining other noninvasive examinations with ETT to improve the diagnostic accuracy for significant CAD. In our study, an attempt was made to address this issue by combining the global CAAS with ETT in 1 diagnostic workup to improve the diagnostics for significant CAD. Our research demonstrated that the diagnostic performance of this new workup (AUC=0.88; *P*<0.001) was significantly better than that of ETT alone (AUC=0.78; *P*<0.001).

Based on the ROC curve analysis, the optimal cut‐off values were suggested to be global CAAS ≤9.22% and DTS ≤−1.12, respectively. When combining these 2 optimal cut‐off values, patients without significant CAD only had a 6% possibility of global CAAS ≤9.22% and DTS ≤−1.12, corresponding to a specificity of 94%. Patients with significant CAD had a 9% possibility of global CAAS >9.22% and DTS >−1.12, corresponding to a sensitivity of 91%. On the other hand, if patients had a global CAAS ≤9.22% and DTS ≤−1.12, the risk of having significant CAD was 85%, corresponding to a positive predictive value. If patients had a global CAAS >9.22% and DTS >−1.12, the risk of having significant CAD was only 8%, corresponding to a negative predictive value of 92%. It is encouraging that the diagnostic procedure for significant CAD was remarkably optimized by the combination of 2 noninvasive methods, which are easily applicable to clinical practice.

Recently, quantification of LV longitudinal strain using 2D‐ST echocardiography was shown to be a sensitive method for identifying significant CAD.^37^ Strain was analyzed in 3 standard apical views.^38^ In each of the apical views, the left ventricle was divided into 6 segments of equal length. Peak systolic longitudinal strain was calculated for the entire U‐shaped length of the LV myocardium (basal, mid, and apical segments of 2 opposite walls) in each view and averaged out to a single value of LV global longitudinal strain.^19^ In our study, the short‐axis view of ascending aorta was divided into 6 segments. Peak CAAS was measured in each of 6 segments and averaged out to a single value of global CAAS. Compared with global longitudinal strain, the measurement of global CAAS was relatively easy and rapid for clinical practice.

### Clinical Implications

The global CAAS analysis based on 2D‐ST echocardiography is a rapid, robust, and reproducible noninvasive method without contraindications and radiation exposure. Based on the present study, 2D‐ST global CAAS may be a feasible and accurate tool for clinicians in the screening of patients with significant CAD. Furthermore, global CAAS may become a supplementary index for risk stratification of CAD, given that it is capable of identifying multivessel CAD.

### Limitations

First, because of the sampling location of ascending aorta, image quality may have been affected by breathing and adjacent structures. Thirty‐seven recruited patients were excluded from the study because the image quality did not reach the standard for accurately tracking and analyzing the vascular image. Second, we used the global CAAS, which was calculated as the mean value of peak CAAS in 6 segments of ascending aorta, to evaluate the changes of elastic properties of ascending aorta. Given the heterogeneity of arterial deformation, it is worth testing whether maximal and/or minimal CAAS are better predictors of coronary stenosis in further investigations. Third, 2D‐ST echocardiography is a semiautomated technology that requires operators to manually draw a line along the inner side of the aortic wall. Thus, this technique is operator dependent, which might limit its wide use in clinical practice. Finally, the consecutive patient enrollment in the study may not have completely precluded selection bias. Further validation and prognostic research are warranted.

## Conclusions

In this study of suspected stable angina patients, global CAAS assessed by 2D‐ST echocardiography at rest was an independent predictor of significant CAD. Furthermore, global CAAS was related to the severity of CAD and capable of identifying multivessel disease. In addition, global CAAS combined with ETT can remarkably improve the diagnostics for significant CAD.

## Sources of Funding

This work was supported by the National Science Fund for Distinguished Young Scholars (81625002); National Natural Science Foundation of China (81470391, 81470389, and 81470389); Shanghai Outstanding Academic Leaders Program (18XD1402400); Shanghai Municipal Education Commission (20152209 and 20172014); Shanghai Science and Technology Committee (15411963600); Shanghai ShenKang Hospital Development Center (16CR3020A and 16CR3034A); National Key Research and Development Program of China (2016YFC1301203); and Shanghai Jiao Tong University (YG2016MS45, YG2015ZD04).

## Disclosures

None.
